# Clinical trials in a dish: cardiometabolic drug development with biological and digital twins

**DOI:** 10.1172/JCI209668

**Published:** 2026-10-01

**Authors:** Debarun Patra, Ibrahim M. Sayed, Ravichandra Venkateshappa, Latha Palaniappan, Tracey McLaughlin, Joseph C. Wu

**Affiliations:** 1 Stanford Cardiovascular Institute and; 2 Division of Cardiovascular Medicine, Department of Medicine, Stanford University, Stanford, California, USA.; 3 Greenstone Biosciences, Palo Alto, California, USA.; 4 Division of Endocrinology, Department of Medicine, Stanford University, Stanford, California, USA.

## Abstract

Preclinical drug development has long relied on animal models to predict safety and efficacy before agents enter human trials, despite critical differences between human and animal model physiology. The withdrawal of rosiglitazone, rofecoxib, and terfenadine due to cardiovascular toxicity exemplifies the translational cost of this mismatch. Alternative, human-based systems enable more accurate modeling of cardiometabolic diseases in a dish; in 2025, the US FDA’s new approach methodologies (NAMs) roadmap authorized the submission of results from human-relevant models. The roadmap encourages utilizing biological and digital twins as part of an integrated, context-specific, fit-for-purpose strategy. A “biological twin” is a human-derived in vitro system that captures the physiology of a patient and can be used to assess potential cardiotoxicity by drug metabolites. A “digital twin” is the computational counterpart trained on clinical drug response results that can further interpret biological twin data at the patient scale and predict pharmacological parameters. NAMs are no longer experimental but are not yet fully validated as replacements for animal models; major challenges remain before they can be effectively incorporated into the cardiometabolic disease drug discovery pipeline. Addressing these challenges head-on is essential for improving drug development and prediction of their cardiovascular safety.

## Introduction

Cardiometabolic disease (CMD) is not a single disease, but an interconnected set of disorders that contribute to cardiovascular complications and heart failure (HF), which account for the highest number of global mortalities (1). Metabolic disorders, such as obesity, type 2 diabetes (T2D), metabolic dysfunction–associated steatohepatitis (MASH), hypertension, dyslipidemia, and stroke, are clinical outcomes of CMD linked with HF (2). Patients are often diagnosed with multiple comorbid conditions that jointly contribute to the cardiovascular-related death toll (3), and the prevalence of such disorders is steadily rising. Metabolic dysfunction–associated steatotic liver disease (MASLD), estimated to affect > 34% of US adults in 2025 and projected to rise substantially by 2050, coexists with obesity, T2D, and hypertension, compounding cardiovascular risk (4).

A typical cardiometabolic drug development program, from preclinical research to FDA approval, can cost over $2 billion and take years (5). Late-stage failures or withdrawal from the market burdens drug developers, regulators, and patients. Roughly 90% of drugs entering trials fail, about half from unanticipated human toxicity or inefficacy (6). Many such failures may be related to a mismatch between human physiology and the biology of the animal models used for drug development — primarily young, inbred rodents and dog species (7). Established animal models have genuine strengths for cardiovascular disease (CVD) and CMD drug development, and large-animal models (e.g., swine), in particular, can recapitulate chronic metabolic disease (8). Nevertheless, differences in drug target expression across species (e.g., nonmammalian targets, such as the microbiome, are entirely different) and differences in drug responses (9, 10) result in poor translation to humans.

A new paradigm may change that picture. Rapid developments in biomedical science, as exemplified by the growing regulatory acceptance and even promotion of new approach methodologies (NAMs) (11, 12) for investigational new drug (IND) submission, are ushering in a brave new world. Researchers can now design effective treatments for cardiometabolic conditions expeditiously and more precisely, bypassing the high failure rates due to toxicities seen with traditional animal-based approaches (9, 13). Although cell-based and in silico alternative models have been developed over the last several decades, the current regulatory momentum and investment is now unleashing their potential.

However, rigorous validation of NAM approaches is required. In parallel, biological twins (human-derived in vitro systems that capture the pathophysiology of patients) are being integrated with digital twins (computational models trained on clinical drug response data) to provide a scalable framework that can detect cardiotoxicity, predict drug exposure, and strengthen regulatory decision-making. For example, artificial intelligence/machine learning-based (AI/ML-based) prediction of drug absorption, distribution, metabolism, excretion, and toxicity (ADMET) and interpretation of in vitro functional readouts and large-scale multiomics data to predict human drug exposure and pharmacodynamic response are outputs of the digital twin (Table 1).

In this Review, we explore how the use of NAMs is transforming drug development and preclinical research in CMD, focusing on two complementary goals. The first goal is to develop and test novel therapeutics for CMDs, and the second is to detect the cardiotoxicity of drugs, including those intended for noncardiac indications, that are unmasked or amplified under cardiometabolic stress.

## Regulatory shift toward fit-for-purpose NAMs

In the history of modern pharmacology research, the “bench-to-bedside” pipeline has overwhelmingly relied on animal models. Since the passage of the Federal Food, Drug, and Cosmetic Act in 1938, the FDA has required that before first-in-human studies, drugs must undergo repeat-dose toxicology studies in two animal species and with evaluation of the effects on multiple organ systems (14). This system was built on the best available science of the last century, but it is increasingly outdated. A main problem is that many animal models were never rigorously validated, and the physiology and genetics of inbred laboratory rodents and other preclinical animal models are considerably different than those of humans (15). This unbridgeable translation gap makes it imperative to develop more human-relevant approaches, particularly for cardiometabolic drug development, given the complexity and long-term nature of these conditions. To that end, there have been multiple regulatory reforms and initiatives from 2022 to 2025 by the FDA and other agencies to move away from animal testing and innovate human-relevant approaches to disease research and drug development that await wider adoption by industry and academia (9, 10, 16, 17).

In December 2022, the FDA Modernization Act 2.0 formally removed the requirement for animal testing in all preclinical drug safety studies (16). Congress explicitly empowered the FDA to accept human-relevant preclinical data (using NAMs such as cell-based assays, organ-on-chip systems, organoids, and computational models) in lieu of exclusive animal studies when supporting IND, new drug application (NDA), and biologics license application submissions. For the scientific community that had spent more than two decades developing human induced pluripotent stem cell–derived (iPSC-derived) cell types, organ-on-chip systems, vascularized human organoid models, and AI/ML models, this was a structural transformation (18–22). The field no longer asks whether to use NAMs; it focuses on how best to use them (Figure 1).

Despite transformation in the regulatory framework, an operational infrastructure for adopting NAMs does not yet exist. In 2024, the Science Board to the FDA’s comprehensive report on NAMs was released (13). It identified persistent gaps in systematic infrastructure for NAM review and explicitly recommended six concrete steps, emphasizing that regulatory acceptance depends on a clearly defined context of use and a fit-for-purpose qualification framework.

Following the translation-focused regulatory changes, in 2024, the FDA Center for Drug Evaluation and Research announced that a human liver-on-chip was accepted into the Innovative Science and Technology Approaches for New Drugs (ISTAND) Pilot Program (23) to assess drug-induced liver injury risk (24). The acceptance of the liver chip signaled that organ-on-chip technologies had advanced from promising research tools to decision-grade regulatory accreditation. In 2025, the FDA laid out its roadmap to reducing animal testing in preclinical safety studies (25). The primary focus will be on monoclonal antibodies and biologics, to be subsequently extended to small-molecule drugs with prioritization of cardiovascular and metabolic compounds. The interlinking developments that the FDA is constructing, including statutory authorization, infrastructure recommendations, a phased roadmap, and a working qualification example, are more than a single policy directive but an updated framework and comprehensive roadmap for the future. For CMD drug development, it is an unprecedented invitation to rebuild the translational pipeline around human biology that can better predict late-stage cardiovascular failures (Figure 1).

## Biological twins

### Technologies.

Biological twin platforms span a complementary spectrum of increasing physiological fidelity and decreasing throughput (Figure 2A). 2D monocultures such as iPSC-derived cardiomyocytes (iPSC-CMs) or iPSC-derived hepatocytes (iPSC-Heps) provide high-throughput efficiency but lack tissue heterogeneity and architecture. Coculture systems (iPSC-CMs/iPSC-Heps with endothelial cells (ECs), fibroblasts, or immune cells) restore paracrine signaling. 3D organoids and engineered heart tissues (EHTs) provide structural maturation and force generation to effectively model disease phenotypes such as fibrosis and diastolic dysfunction. 3D bioprinting extends this toolbox by spatially depositing patient-derived cardiac, vascular, and hepatic cells into architecturally defined, perfusable tissues with reproducible geometry, thereby enabling scalable fabrication of cardiometabolic biological twins (26–28). While bioprinting provides anatomical geometry, static cultures lack dynamic physiology. Bioreactors advance in vitro disease modeling by combining fluid flow, mechanical stretch, and real-time monitoring to induce scalable tissue maturation (29, 30). Organ-on-chip systems introduce fluid flow, shear, mechanical cues, efficient oxygen exchange, and other factors that mimic the human microenvironment, whereas multiorgan microphysiological systems (MPSs) connect tissues to capture interorgan metabolite relay and allow study of drug response. CMD involves interorgan crosstalk, which can be modeled using an MPS connecting the gut, heart, liver, and adipose tissue to assess oral drug absorption, first-pass hepatic metabolism, and downstream cardiac exposure (31–33). Fidelity rises across this spectrum, but so do cost, variability, and standardization burden. An appropriate platform should be dictated by the context of use, not by maximal complexity.

An example of a biological twin for CMD is the metabolically stressed hepatic model (primary hepatocytes or iPSC-Heps), which can be coupled to cardiomyocytes in an MPS to reconstruct the liver/heart signaling axis (34, 35). Another example of a biological twin to assess diabetic cardiac complications is derived from a T2D iPSC-vascularized cardiac organoid under inflammatory stress and hyperglycemic conditions that can be placed on a microelectrode array (MEA) (36, 37). Additionally, a blood vessel-on-chip under lipid and inflammatory stress can serve as the biological twin of microvascular dysfunction (38, 39). Unlike generic assays, biological twins capture patient-specific biology, genetics, and metabolic context, making them powerful platforms to capture drug responses.

### Cell and organoid villages.

The “village-in-a-dish” approach allows us to study variables of genetic diversity, sex, age, and immunity in the population by pooling, culturing, and differentiating multiple donor iPSC lines in a single dish, then computationally assigning each cell to its donor using single-cell genotyping (Figure 2B) (40, 41). As the iPSC lines share the same medium, batch, and microenvironment in culture, this approach minimizes technical variation and preserves line-specific genetic and epigenetic effects. Organoid villages extend this principle from monolayers to 3D tissues, enabling cellotype–phenotype studies that link genetic and epigenetic effects to function in a tissue-relevant context and can corroborate GWAS and expression quantitative trait locus signals with modest cohorts (42). This capability is directly relevant to CMD drug development. CMD modeling with the village concept allows differentiation of iPSCs into multitissue organoids, including heart, liver, kidney, adipose, and blood vessels. For example, a panel of 68 donor iPSC lines was differentiated into hepatocytes and adipocytes to study the effect of a common noncoding variant at 1p13 (rs12740374) in CMD (43). The investigators found a clear association between the variant and a lipid phenotype in hepatocytes, characterized by higher expression of CELSR2, PSRC1, and SORT1. Mechanistically, increased SORT1 expression was associated with lower LDL-cholesterol, explaining SORT1’s protective effect on LDL-cholesterol secretion and myocardial infarction risk.

Cell/organoid villages also allow testing of drug efficacy and toxicity across diverse donors simultaneously. The outcome represents a remarkable shift from single-patient–scale twins toward population-scale prediction and the provision of the genetically anchored data that population-scale digital twins require. However, there are potential caveats to the implications of this technology, as the lines proliferate differently and can competitively overgrow, so accurate representation is essential for disease modeling (44).

### Identifying hidden drug toxicities in CMDs.

Testing the long-term safety of CMD drugs in healthy animals commonly misses the cardiovascular toxicities of such compounds in human patients (45). Since CMDs are combined comorbid conditions that affect the liver, gut, adipose, kidney, vasculature, and heart, they create an altered environment that modulates a drug’s effect (46).

For example, MASLD/MASH independently accelerates the risk of CVD events by two- to threefold (47). The endocrine basis of the liver/heart axis has been well characterized and clinically established, and it works bidirectionally (48, 49). Whereas the MASH liver drives cardiac remodeling, reduced cardiac output in HF combined with reduced ejection fraction causes hypoperfusion and ischemia in the liver (50, 51). Elevated right-sided filling pressures produce congestive hepatopathy, which impairs hepatic drug metabolism and synthetic function (52, 53). Moreover, hepatokines alter heart function, with FGF21 exerting an antifibrotic effect and improving metabolic parameters (54), SAA1/4 amplifying cardiac inflammation (55), and Factor XI activating cardioprotective BMP/SMAD1/5 signaling and protecting against HF with preserved ejection fraction–induced (HFpEF-induced) fibrotic remodeling (56). Hepatocyte-derived extracellular vesicles carrying miRNAs are also reported to regulate cardiac lipid metabolism and mitochondrial function (57). These factors indicate the possibility of liver-derived cardiotoxic mediators that are not measured by current standard preclinical assays.

Finally, decreased CYP450 expression, depleted mitochondrial function, and accelerated lipid deposition can dampen the overall drug metabolism potential of the MASH liver (58). Multiple studies have shown that CYP3A4 expression and enzymatic activity are reduced in the setting of MASH, implying variability in drug exposure across the polypharmacy-dense CMD and CVD landscape (59, 60). Indeed, a drug that is metabolized into a cardio-inert compound in a healthy liver can, in MASH, be only partially metabolized, accumulating in the heart as an alternate metabolite that blocks the human ether-à-go-go–related gene (hERG) channel activity and causes arrhythmia (61). Similarly, in the healthy liver, the gastroprokinetic drug cisapride is converted to norcisapride, but in the setting of hepatic CYP3A4 deficiency, the unmetabolized parent compound escapes the hepatic circulation and flows directly into the connected cardiac compartment (62).

These types of hidden toxicities can be tested for in a liver/heart MPS with a potential disease-state human liver component (Figure 3A) (63). Oleaga et al. developed a microfluidic platform that includes a coculture of iPSC-CMs and primary hepatocytes and assessed the platform’s ability to detect liver metabolite–mediated cardiac toxicity of two known drugs: cyclophosphamide and terfenadine (34). Both drugs showed metabolite-mediated cardiotoxic effects. Another MPS including four organs (heart, liver, muscle, and neurons) was developed to assess the effects of five drugs (atorvastatin, valproic acid, doxorubicin, *N*-acetyl-m-aminophenol, and acetaminophen) and a control compound on different organs. Liver toxicity was reported for all tested drugs except for *N*-acetyl-m-aminophenol, consistent with the literature; cardiotoxicity was mainly observed with doxorubicin (64). A cardiac-only platform treated with propranolol demonstrated a dose-dependent reduction in beating rate of 10% to 40%. By contrast, a cardiac-liver platform treated with the same drugs did not show a significant increase in heart rate due to drug metabolism by hepatocytes that produced metabolites that were inactive or less active in the heart (63, 65). These studies illustrate the requirements for a multitissue-integrated in vitro system to study drug response, efficacy, and toxicity of CMD candidate drugs. Notably, most MPS run for days to a few weeks, so chronic toxicity currently can only be inferred rather than directly measured.

### Arrhythmia risk assessment.

Drug-induced arrhythmia is a major cardiac safety concern for all drug development programs. CMDs amplify the proarrhythmic susceptibility in patients (66, 67). For example, T2D induces cardiac electrical and structural remodeling, altering both expression and function of ion channels, compromising repolarization reserve, and reducing the threshold for drug-induced arrhythmias (36, 66). Another example is hypertension, which drives left ventricular hypertrophy that results in significant prolongation of action potentials, primarily due to reduced potassium current, ultimately leading to increased dispersion of repolarization and a substrate for reentry (67). These effects suggest that drug-induced ion channel perturbation operates with a far narrower safety margin in patients with CMDs, which may be better evaluated in NAMs than by testing in healthy animal models. Indeed, the comprehensive in vitro proarrhythmia assay (CiPA) initiative, a blinded multisite study of iPSC-CMs on MEAs, correctly stratified the torsadogenic risk of 28 reference drugs (68). Another study on human in silico ventricular models predicted clinical proarrhythmia for 62 compounds with approximately 89% accuracy (69). A comprehensive 26-drug assessment and blinded cross-site and international multisite study demonstrated the reproducibility and predictive accuracy of the iPSC-CMs, supported by consensus best-practice recommendations (68, 70, 71). A “clinical-trial-in-a-dish” also reproduced the clinical QT prolongation of two QT-prolonging drugs tested in donor iPSC-CMs (72). Cardiac NAM platforms have prospectively predicted the proarrhythmic risk of challenging agents, including vanoxerine and repolarization-balanced (prolonging plus shortening) drug combinations (73–75). The combination of 2D/3D iPSC-CM platforms, MEA readouts, and in silico action potential modeling under cardiometabolic stress thus provides a validated route to arrhythmia risk assessment.

As noted above, metabolic failure allows intact, hERG-active parent compounds to escape hepatic degradation, bioaccumulate, and directly interact with the downstream myocardium (34). In the heart, these parent drugs physically block the α subunit of the voltage-gated potassium channel, thereby delaying phase 3 cardiac repolarization, prolonging the QT interval, and creating the functional substrate for ventricular arrhythmias (76). Physically coupling human-derived hepatocytes, particularly from metabolically stressed liver (mimicking MASH), and cardiomyocytes within an MPS enables experimental interrogation of the interplay between hepatic first-pass metabolism and off-target cardiotoxicity (Figure 3B) (77).

### Obesity and T2D.

Both obesity and T2D drive lipotoxicity that substantially affects vascular and cardiac functions. Obesity shifts the heart’s bioenergetic landscape from glucose oxidation to fatty acid overutilization (78). In turn, the oxidative by-products sensitize cardiac cells to drug-induced injury (79). The risk of dyslipidemia is also heightened in obese patients, further elevating proinflammatory lipid mediators (80). In combination, these stress factors impair endothelial NO synthase activity, which causes endothelial dysfunction that can progress to coronary disease (81). Testing drugs in iPSC-CMs/vascularized cardiac organoids exposed to lipotoxic stress or in an MPS incorporating steatotic hepatocytes is therefore an essential strategy to identify cardiotoxic risk (34). Advanced in vitro models can successfully unmask cell-intrinsic pathologies underlying lipid-induced hERG trafficking defects and mitochondrial bioenergetic collapse under drug challenge, which are broadly absent in preclinical animal screens (82–84).

In T2D, conditions such as hyperglycemia, insulin resistance, advanced glycation end product buildup, and chronic inflammatory stress result in impaired vasodilation and activation of proinflammatory and profibrotic cascades in the heart (85). Baseline endothelial biology is altered in T2D, making drug response studies in T2D iPSC-ECs especially clinically significant (86, 87). Results from the Progression of Early Subclinical Atherosclerosis cohort have now confirmed that impaired coronary microvascular function is detectable in middle-aged asymptomatic individuals with CMD risk factors before any obstructive coronary disease is apparent (88). It is therefore essential that cardiometabolic drugs be tested in this early-pathology window (Figure 3C).

### Vascular models.

Human 3D models or MPS platforms allow for precise temporal and spatial modeling of subclinical hallmarks of atherosclerosis, including endothelial activation, fluid shear stress mechanotransduction, and localized monocyte transmigration prior to advanced plaque deposition (89–91). ECs, vascular smooth muscle cells (VSMCs), and pericytes can be integrated and placed under metabolic stress prior to testing a drug to allow vascular safety assessment (38). Readouts include impedance sensing, NO release, adhesion protein (e.g., e-selectin, ICAM-1) expression, or multiomics assays depending on the context (21, 92). Vila Cuenca et al. generated an iPSC-derived vessel-on-chip incorporating ECs and VSMCs in a fibrin hydrogel microenvironment (93). This produced a self-organized, lumenized structure with perfusable microvascular networks that is responsive to vasoactive stimulation. Moreover, VSMC responses to drug treatment were quantifiable by measuring intracellular Ca^2+^ release kinetics. Another reproducible perfused vessel-on-chip platform maintained under physiological shear demonstrated well-characterized inflammation biology, including TNF-driven morphological remodeling (39). A multilineage cardiovascular organ chip generated by Mozneb et al. showed further advancement of this architecture by incorporating iPSC-CMs and iPSC-ECs on separate microfluidic channels under active fluid flow and rhythmic biomechanical stretch (35). This platform demonstrated functional stability with endothelial barrier permeability and revealed cardiotoxicities of a vascular-disrupting tyrosine kinase inhibitor (35). Vascular NAMs are also being used to test whether drugs can successfully rescue endothelial function before structural vascular disease becomes irreversible.

### HF with preserved ejection fraction.

HFpEF is characterized by myocardial stiffness with fibrosis and hypertrophy, diastolic dysfunction with prolonged relaxation and impaired calcium handling, increased ROS production, and mitochondrial dysfunction with decreased ATP content (94). The PROMIS-HFpEF study found coronary microvascular dysfunction in 75% of HFpEF patients, identifying microvascular dysfunction as the dominant coronary phenotype in this population (95).

Although multiple rodent models exist for studying HFpEF, they have limitations (96–98). These models depend on artificial multihit interventions and do not reproduce the comorbidity-driven coronary microvascular inflammation that defines human HFpEF (97). Moreover, species-specific differences in myocardial stiffening, titin isoform expression, and NO synthase kinetics mean that animal results rarely translate successfully to human clinical outcomes (99).

Disease-conditioned NAMs offer an appealing solution to capture the HFpEF mechanism that is largely inaccessible in vivo. Cardiac organoids exposed to (a) angiotensin II and endothelin I to mimic hypertension, (b) IL-1β and IFN-γ to mimic obesity-related inflammation, or (c) high glucose and insulin deprivation to mimic diabetes showed diastolic dysfunction with prolonged relaxation, increased ROS, and reduced oxygen consumption, suggesting that these models can recapitulate the pathophysiology of HFpEF (36).

Notably, iPSC-CMs share limitations with other iPSC-derived cell types and organoid models, including an immature, fetal-like phenotype, a lack of adaptive immune cells, and an inability to generate the chronic, multiorgan comorbid milieu that drives human HFpEF. These constraints differ in kind from those of animal models, which fail to capture human-specific cellular electrophysiology and genetic diversity (100). However, recent advances have improved protocols for iPSC-CM maturation and the development of complex organoids with microvasculature and immune cells (101, 102). In a human EHT model of lipotoxic/diabetic cardiomyopathy, treatment with the SGLT2 inhibitor empagliflozin reduced mitochondrial injury, endoplasmic reticulum stress, and cell death and improved contractile function (103), supporting the utility of such biological twins for HFpEF drug testing.

### Hypertension.

Hypertension causes pressure overload, induces concentric left ventricular hypertrophy, activates the neurohormonal axis, and promotes myocardial fibrosis via TGF-β, endothelin-1, and aldosterone signaling (104). Drugs acting on these pathways (e.g., angiotensin receptor-neprilysin inhibitors, novel soluble guanylate cyclase stimulators, and mineralocorticoid antagonists) target the remodeled myocardium, which has altered mechanical stiffness, calcium handling, and electrophysiology (105, 106). iPSC-derived EHTs under mechanical stretch can be used to simulate chronic pressure overload, recapitulating hypertrophic remodeling to detect drug effects in the disease state (Figure 3C) (107, 108).

### Organ-on-chips for dynamic time-resolved exposure modeling.

It is important to assess the pharmacokinetic and pharmacodynamic (PK/PD) profile of a target drug, including its bioavailability, volume of distribution, metabolism, expected metabolite profiles (phases I and II), and clearance, especially in cardiometabolic stress conditions (109). To achieve this, Edington et al. developed an interconnected MPS of four cell types (gut, liver, lung, and endometrium), a seven–cell type MPS (the previous cells plus heart, pancreas, and brain), and a 10–cell type MPS (the previous seven–cell type system plus muscle, skin, and kidney) (110). Other MPSs include primary human intestinal epithelium using biopsy-derived organoids and inflamed gut epithelium in the inflammatory microenvironment (10). In a gut-on-chip model, a mechanical force is applied to mimic peristaltic-like intestinal movement (111). The gut-on-chip can be used to provide precise measurements of drug exposure (112). The liver-on-chip features controlled fluid flow and mechanical shear resembling blood flow through the multicellular liver compartment comprising hepatocytes, Kupffer cells, ECs, and stellate cells (113). To mimic the MASH microenvironment, Slaughter et al. developed a chip model including primary human hepatocytes and adipocytes (114). In addition, iPSC-CMs have been successfully incorporated into the chip model to measure drug effects on heart electrophysiology and contractility (37). These platforms have been used to test agents such as the SGLT-2 inhibitor empagliflozin; in iPSC-CMs, it abolished hyperglycemia-induced hypertrophy, normalized calcium handling, and increased Na^+^ currents (115). Lastly, multiorgan MPSs linking kidney, liver, and heart organoids in series offer a path for better prediction of PK/PD (116, 117).

## Digital twins

Whereas the biological twin generates the context-specific readouts from experiments that a generic standard assay cannot, the digital twin is the computational part that interprets such data to make patient-scale predictions. The digital twin concept originated in the physical sciences more than 50 years ago (118). In the last two decades, the concept has been applied to biomedical research and integrated with the biological twin. The full realization of these two complementary approaches will substantially strengthen context-specific NAM strategies.

### Predicting exposure and cardiovascular risk at patient scale.

In medicine and healthcare, a digital twin is more than a single predictive model (119). It is a continuously updated computational replica of a patient or a virtual population that integrates multiscale data, including molecular and cellular readouts and tissue, organ, and clinical measurements, to forecast disease trajectory and therapeutic response (120, 121). There are two complementary modeling philosophies: mechanistic digital twins encode known physiology and pharmacology (e.g., physiologically based pharmacokinetic [PBPK] modeling and in silico electrophysiology), whereas data-driven and generative digital twins learn the mapping between biological twin readouts and clinical outcomes directly from data (120, 122). For CMD drug development, the biological twin provides disease-specific, patient-derived measurements that parameterize and constrain this replica, e.g., the organ-on-chip and organoid data that the digital twin uses to make individualized predictions of drug exposure, cardiotoxicity, and cardiovascular risk. The digital twin prediction can be done at the patient scale (e.g., using a twin parameterized on one individual’s biological twin and clinical data to forecasts that person’s drug exposure and cardiotoxicity) or at the population scale (e.g., diverse donor iPSCs or virtual patient cohorts can be sampled across genetic and physiological variability to predict drug responses and flag susceptible subgroups). This approach offers a transformative opportunity to predict human-relevant drug exposure, organ-specific concentrations, and metabolic clearance in CMD, which no single experimental platform can replicate in isolation.

Digital twins also mature in stages, a progression that clarifies where current cardiology models sit (123). The earliest “static” twins are data-driven mathematical models built from fixed clinical inputs — exemplified in cardiology by image-based heart models used for procedural planning (124). By comparison, “progressive” and “operational” twins are continuously updated by streaming data and refined through reinforcement learning (123). The latter approach allows the model to define each individual’s physiological “normal,” flag deviations as emerging disease, and forecast the outcome of an intervention. A key application for drug development is the virtual patient, in which a calibrated twin can be exposed in silico to many candidate drugs, doses, and regimens to forecast efficacy and adverse effects before any human exposure, reducing the cost and ethical burden of testing in real patients. For CMD drug development, the integration of multiscale data from molecular to tissue, organ, and whole-body physiology will be required, which the biological twin is designed to supply (125).

### AI and ML integration.

The most forward-looking capability is in silico intervention using AI and ML platforms (126). Generative digital twins can simulate “what if” scenarios by predicting how a virtual patient would respond to an untested drug, dose, or combination before any experiment is performed (122). This mirrors advances in AI foundation models trained on large biological datasets, which can now predict generalized responses to entirely new, unseen inputs. Recent studies demonstrate the neural activity of digital twins that forecasts responses to novel stimuli (127). The 2025 FDA roadmap highlights in silico NAMs as critical complements to experimental MPSs, leveraging machine learning approaches (13, 25). This has stimulated the rapid development of mature digital twin modalities, including models that are trained on iPSC-CM MEA data, multi–organ-on-chip ADMET readouts, secretory protein profiles, and clinical adverse event databases (128). These models can flag the cardiotoxicity and exposure liabilities at population scale for novel chemical entities before a single experiment is run, essentially enabling computational triage (129, 130). Because inflammation is a central driver of CMDs, incorporating immune dynamics as “immune digital twins” will be important for forecasting drug responses (131). This vision will require rigorous, prospective validation against clinical outcomes before making in silico predictions.

Integrating these biological and digital twin components into a single, end-to-end workflow will be critical for cardiometabolic drug development (Figure 4). The process begins with the selection of a defined, diverse patient population and a disease-conditioned biological twin experiment. An example is the vascularized liver/heart MPS built from patient-derived cells, whose functional readouts calibrate the digital twin. The calibrated disease model then combines AI/ML-based ADMET prediction, cardiac electrophysiology simulation, and PBPK drug exposure modeling to generate patient-scale risk predictions for cardiotoxicity. Each prediction guides the next biological twin experiment and is anchored to a regulatory context-of-use checkpoint that defines the specific safety question the platform is qualified to answer. This allows researchers to extract morphological features of cardiac, vascular, and liver cells/organoids using high-content imaging together with multiomics and then to integrate them with publicly available clinical datasets to train and validate a prediction model. Finally, the validated model can be applied to high-throughput screening of large compound libraries to discover mechanisms of action, repurpose drugs, and optimize leads (Figure 4). To summarize, a digital twin can convert disease-specific, human-relevant measurements into actionable, population-level predictions that feed directly into regulatory decision-making.

## Conclusions, challenges, and opportunities

A disease-conditioned human-derived cardiac, hepatic, and vascular platform can resemble the CMD patient’s pathophysiological state in vitro, serving as a biological twin of the human. A digital twin can scale those signals to the patient population level and predict the pharmacological response. We assert that integration of biological and digital twins has the potential to close the translational gap in CMD drug development. However, widespread adoption of NAMs in the evolving regulatory landscape demands standardized operating procedures, prospective clinical validation models, and ISTAND qualification of multiorgan platforms (Table 2). Despite growing progress, significant hurdles remain before NAMs can be fully adopted, which will first augment animal models and ultimately become the primary tool for predicting preclinical safety, a process that requires a precise context of use (132).

### Context of use.

Although NAMs are approved for regulatory submission, regulators continue to find insufficient the context of use in submitted NAMs, a key technical deficiency for regulatory approval (133). An example is the liver-heart MPS, which can measure hepato-cardiac toxicity of a given drug (134). Researchers using the liver-heart MPS as a NAM must answer the following safety questions: Does it provide evidence of thresholds for positive and negative results? What other NAM data have been used in the same context to answer safety questions? How can different NAMs be integrated into a weight-of-evidence framework (132)? In the case of a liver-heart MPS, the context of use can be defined as the detection of metabolite-dependent cardiotoxicity in a metabolically stressed hepato-cardiac system. Another measure is the quantification of endothelial dysfunction risk under hyperglycemic stress. Without reaching this level of specificity, NAM data generated at great expense may not be ideal for regulatory decision-making.

### Circadian rhythm of the liver/heart axis.

More than 15% of the transcriptome for the human liver is linked with diurnal oscillation, which governs glycogenesis, fatty acid oxidation, and, most importantly, the xenobiotic detoxification cycle across the 24-hour circadian clock (135, 136). CYP enzymes reach peak activity during the rest phase (137). In MASH patients, circadian clock gene expression is disrupted by metabolic imbalance, inflammation, and unregulated feeding patterns, which also affect drug metabolism potential. Hepatic and peripheral insulin resistance increase at night in MASLD patients with greater insulin clearance and less insulin release (138). Importantly, the pharmacological aspects (e.g., PK and PD) of many drugs in humans are governed by the circadian clock, making it critical to consider the variability in drug metabolism, detoxification, and efficacy (139, 140). Recently, a validation study found that in vitro primary human liver platforms possess robust, autonomous peripheral clocks that maintain the rhythmic, 24-hour transcriptomic oscillation of > 300 genes regulating lipid metabolism, cytochrome P450 activity, and pathogen-induced inflammatory signaling (135). When integrated with automated fluidic microformulators that programmatically pulse physiological endocrine cues, the platform could sustain long-term chronopharmacological cycles. This programmatic control allows investigators to map toxicity and drug efficacy profiles under both healthy and metabolically disrupted states.

### Reproducibility and standard operating procedures.

Standardized protocols are required to compare data reproducibility across laboratories and to benchmark reference compounds to aid regulator decision making (141). Specific barriers include the intrinsic variability in donor iPSC lines, arising from clonal/epigenetic states, as well as varying differentiation protocols between laboratories. Due to these differences, the same nominal assay can yield divergent phenotypes across sites. Isogenic and multiline panels and interlaboratory ring trials with shared reference compounds are therefore essential (142, 143). In March 2024, the Interagency Coordinating Committee on the Validation of Alternative Methods reported a reference framework for assessing NAM submission, specifically identifying reproducibility, quality control, data integrity, and information transparency as the central barriers to NAM regulatory acceptance (144). To that end, the field should converge on a single, fully reported protocol producing a defined, reproducible phenotype for regulatory comparison, rather than relying on each laboratory to pursue a moving “maturity” target.

### Multiorgan culture media.

Growing organs in a dish is possible today with the great advances in relevant technologies and engineering, and researchers have developed MPSs representing multiple organs (e.g., heart, liver, blood-brain barrier, vessel, adipose, gut, and kidney) (31). The challenge here is for the NAM research community to generate a “universal medium” to modernize CMD modeling.

### The path forward.

The path for cardiometabolic NAM researchers is quite actionable under the following three steps. (a) There is a timely requirement for prospective validation datasets comparing NAM predictions for approved drugs against known clinical outcomes. Investigators can first validate NAM performance retrospectively using drugs with established cardiovascular safety profiles and then test novel compounds to generate the prospective evidence base that regulators require to evaluate new NAM-based readouts. (b) MPS developers focusing on the multiorgan cardiometabolic system should pay attention to the ISTAND qualifications for liver-heart and kidney-liver-heart platforms. This is especially critical if the goal is to capture the pharmacological response of MASH and T2D. (c) Further development of digital twins is essential for NAM success. MPS assay readouts can be integrated into models to generate the calibrated in vitro to in vivo extrapolation parameters. This approach is necessary to translate organ-on-chip drug exposure data into human plasma and tissue concentration predictions under metabolic disease conditions (Figure 4).

Overall, the goal is not to replace all animal testing immediately. Instead, the field needs to ensure that all CMD drug programs henceforth advance to first-in-human studies after being evaluated in a human-relevant, disease-conditioned biological context that the intended patient population inhabits.

## Conflict of interest

JCW is a cofounder of and scientific advisor to Greenstone Biosciences.

## Funding support

This work is the result of NIH funding, in whole or in part, and is subject to the NIH Public Access Policy. Through acceptance of this federal funding, the NIH has been given a right to make the work publicly available in PubMed Central.

- NIH grants R01 HL145676, R01 146690, R01 HL150693, R01 HL178528, UG3 AG097135, and UM1 TR006031 (to JCW).
- Stanford Medicine ADVANCE program fellowship (to DP).
- American Heart Association fellowship 26POST1562083 (to DP).
- Kleberg Foundation Medical Research Grant Program (to JCW).
- Gootter-Jensen Foundation (to JCW).

## Figures and Tables

**Figure 1:**
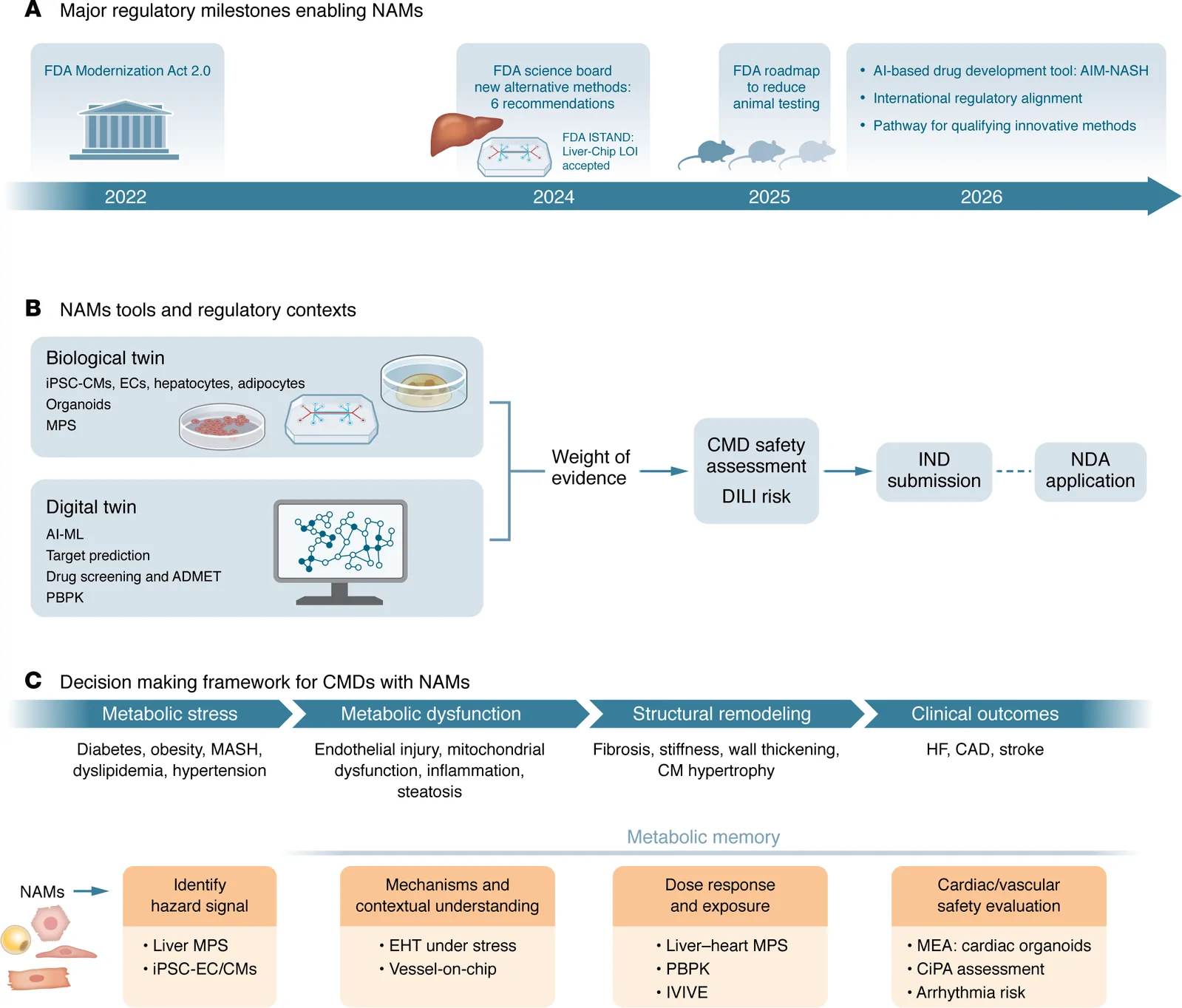
Regulatory milestones enabling NAMs and the decision-making framework for cardiometabolic drug development. (**A**) Timeline of key regulatory milestones from 2022 to 2026, including the FDA Modernization Act 2.0, ISTAND Liver-on-Chip Letter of Intent (LOI) acceptance, FDA Science Board six-point NAM recommendations, the 2025 FDA Roadmap to Reducing Animal Testing, and the 2026 year-one progress and milestone. (**B**) NAM tools for regulatory submission. Biological twin platforms (iPSC-CMs, ECs, hepatocytes, adipocytes, multicellular organoids, and MPSs) and digital twin platforms (AI/ML ADMET, drug screening, PBPK, and target prediction) provide a weight-of-evidence package supporting CMD safety assessment, drug-induced liver injury (DILI) risk evaluation, and IND/NDA submissions. (**C**) The NAM-based decision framework has been mapped across CMDs, from metabolic stress to metabolic dysfunction and structural remodeling to clinical outcomes. NAM platforms are assigned to each stage: hazard identification, mechanistic understanding, dose–response and exposure modeling, and cardiac/vascular safety evaluation. CAD, coronary artery disease.

**Figure 2:**
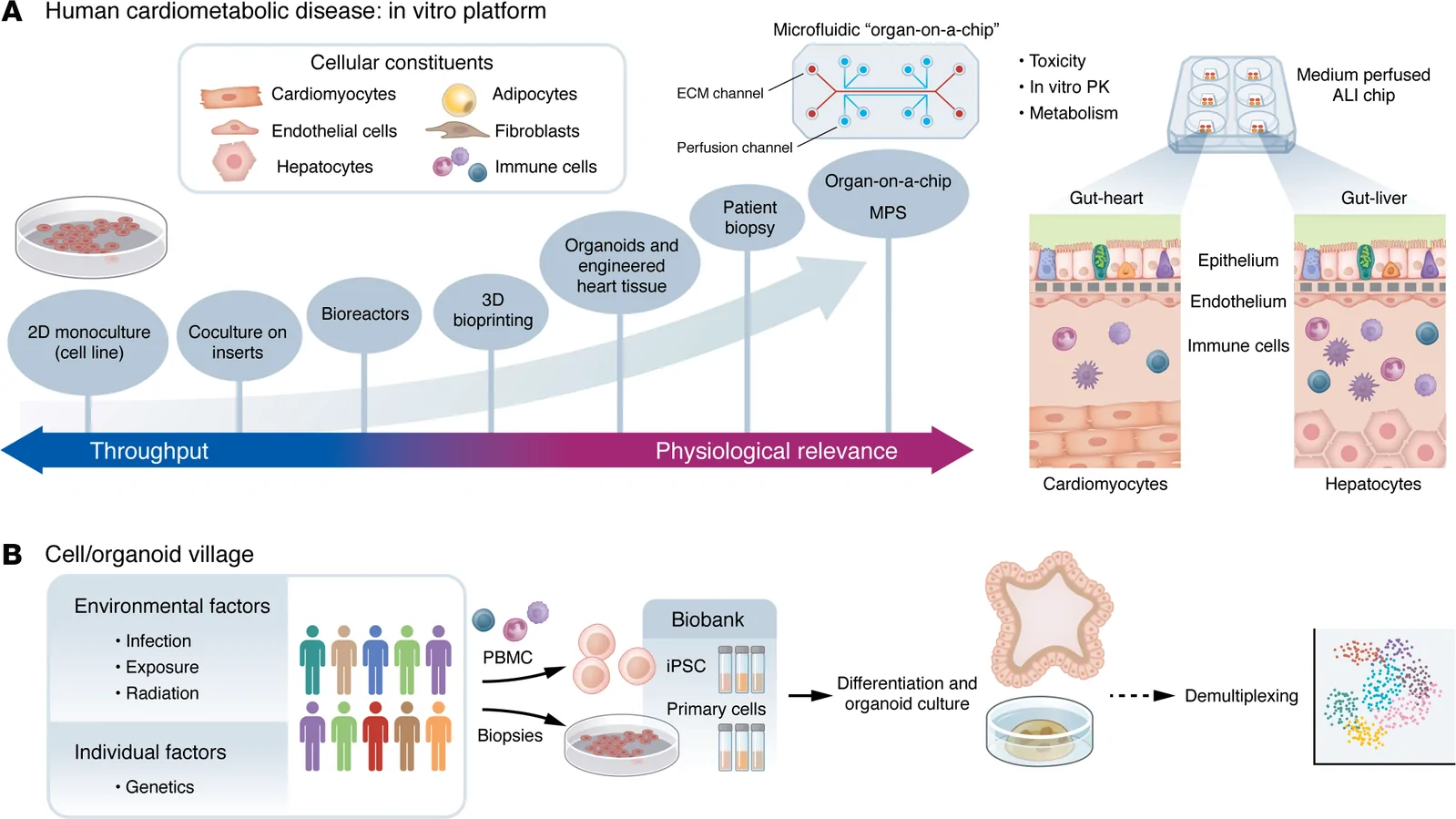
Biological twin platforms for CMD modeling from patient to population level. (**A**) Platforms are arranged along axes of throughput and physiological relevance from 2D monoculture and coculture inserts through bioreactors, 3D bioprinting, organoids, and EHTs to organ-on-chip and fully integrated MPSs. Cellular constituents include cardiomyocytes, ECs, hepatocytes, adipocytes, fibroblasts, and immune cells from iPSCs or primary patient sources. Insets show microfluidic chip architecture and gut-heart and gut-liver multiorgan configurations relevant to oral drug absorption, first-pass hepatic metabolism, and downstream cardiac exposure assessment. ALI, air-liquid interface. (**B**) Organoid village strategy for population-scale biological twin modeling. Patient-derived iPSCs and primary cells obtained from PBMCs and tissue biopsies are stored in a multidonor biobank representing diverse genetic backgrounds and CMD states. These are differentiated into organ-specific organoids and cultured as mixed-donor organoid villages. Single-cell transcriptomics and computational demultiplexing enable population-scale drug response profiling that captures interindividual variability in a single experimental run.

**Figure 3:**
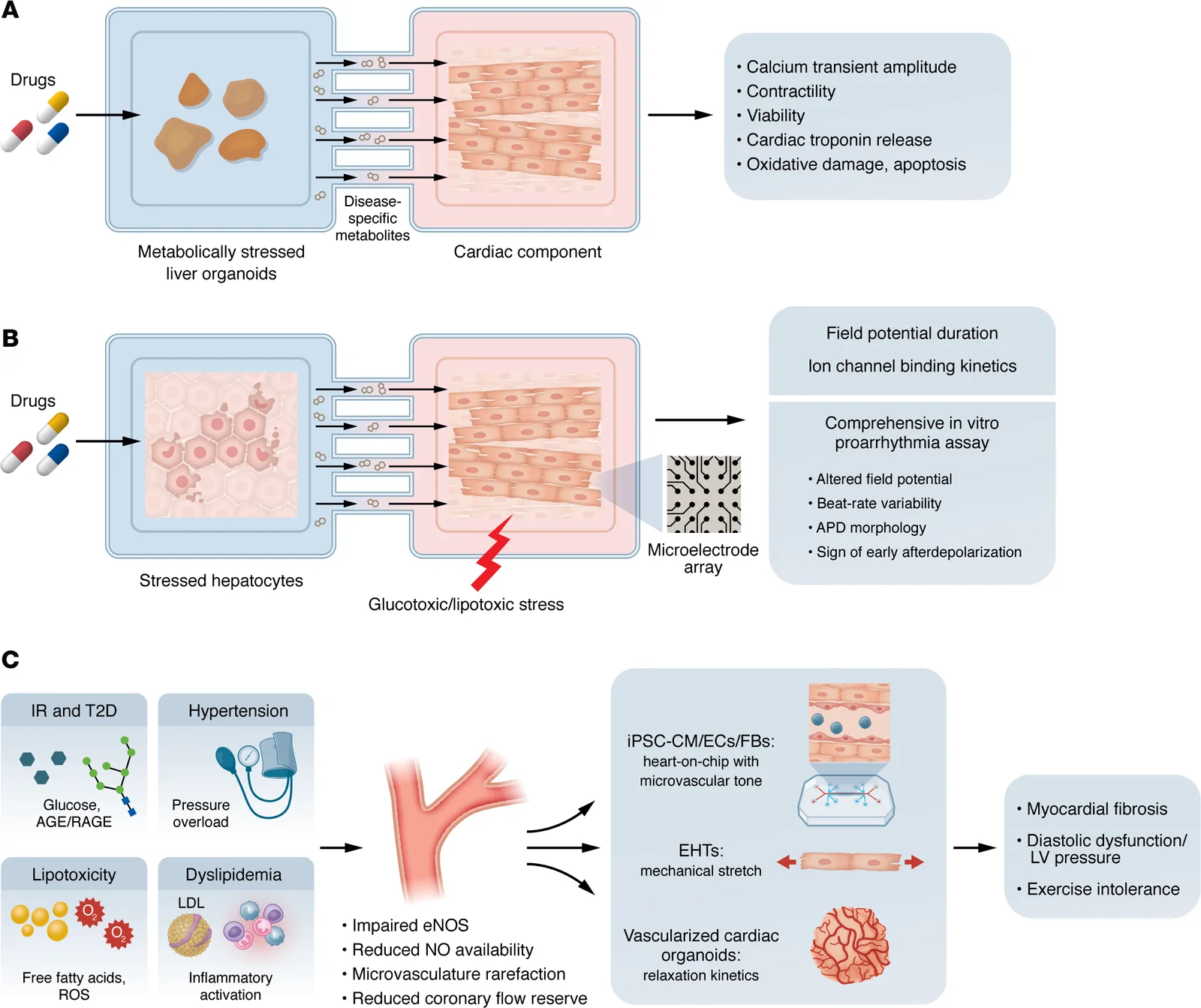
Detecting cardiotoxicity, proarrhythmia risk, and microvascular dysfunction under cardiometabolic stress. (**A**) Liver-heart MPS for metabolite-mediated cardiotoxicity detection. Drugs administered to metabolically stressed hepatic organoids generate disease-specific metabolites delivered via microfluidic flow to the cardiac compartment. Cardiac readouts include calcium transient amplitude, contractility, viability, troponin release, and oxidative injury markers. (**B**) Integrated heart/liver axis arrhythmia risk assessment combining MEA recording with the CiPA framework. Drugs administered to MASH-stressed hepatocytes generate disease-specific metabolites delivered to iPSC-CMs simultaneously exposed to glucotoxic and lipotoxic stress. MEA captures CiPA readouts of altered field potential, beat rate variability, action potential duration (APD) morphology, and early afterdepolarization. In silico action potential modeling using ion channel binding kinetics integrates with MEA data to generate mechanistic proarrhythmia risk scores, capturing metabolite-mediated and disease state–amplified arrhythmia risk beyond the resolution of standard hERG testing. (**C**) Four converging disease processes — insulin resistance (IR)/T2D (advanced glycation end product/receptor for advanced glycation end products [RAGE] accumulation), lipotoxicity (free fatty acids, ROS), hypertension (pressure overload), and dyslipidemia/inflammation (LDL, cytokines) — impair eNOS coupling, reduce NO bioavailability, and cause coronary microvascular rarefaction and reduced coronary flow reserve. NAM platforms address the following endpoints: heart-on-chip (iPSC-CM/ECs/fibroblasts [FBs]) for microvascular tone, EHT under mechanical stretch for hypertensive remodeling, and vascularized cardiac organoids for diastolic relaxation kinetics. Clinical translational outputs include myocardial fibrosis, diastolic dysfunction, and exercise intolerance (146–148). LV pressure, left ventricular pressure.

**Figure 4:**
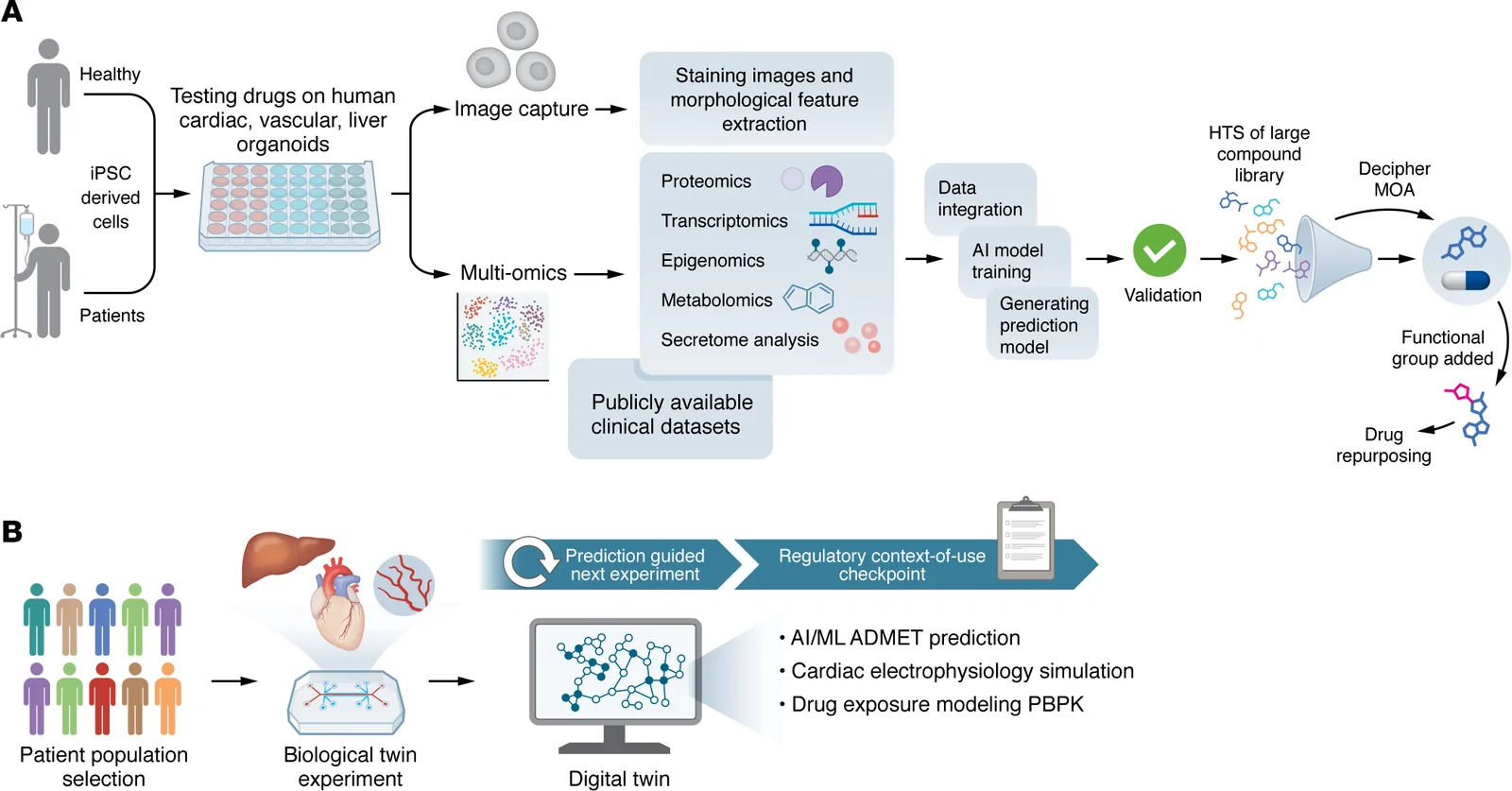
Integrated biological and digital twin framework for cardiometabolic drug safety and regulatory submission. (**A**) AI/ML-driven digital twin pipeline. iPSC-derived cardiac, vascular, and liver organoids from both healthy donors and cardiometabolic patients are exposed to drug candidates. Morphological feature extraction from high-content staining images and multiomics data streams — including proteomics, transcriptomics, epigenomics, metabolomics, and secretome analysis — are integrated with publicly available clinical datasets to train AI prediction models. These models enable high-throughput screening (HTS) of large-compound libraries, mechanism-of-action (MOA) deconvolution, lead optimization with functional group addition, validation, and drug repurposing, collectively bridging organoid-scale phenotyping to patient-scale pharmacological prediction. (**B**) A defined patient population anchors disease-conditioned biological twin experiments across liver-heart MPS, vascularized cardiac organoids, and coronary microvascular chips. Functional readouts feed three digital twin components (AI/ML ADMET prediction, cardiac electrophysiology simulation, and PBPK modeling) to generate a calibrated patient-scale pharmacological prediction. Two iterative outputs are produced: prediction-guided experimental refinement and a regulatory context-of-use checkpoint for IND/NDA submission through the ISTAND framework.

**Table 1:**
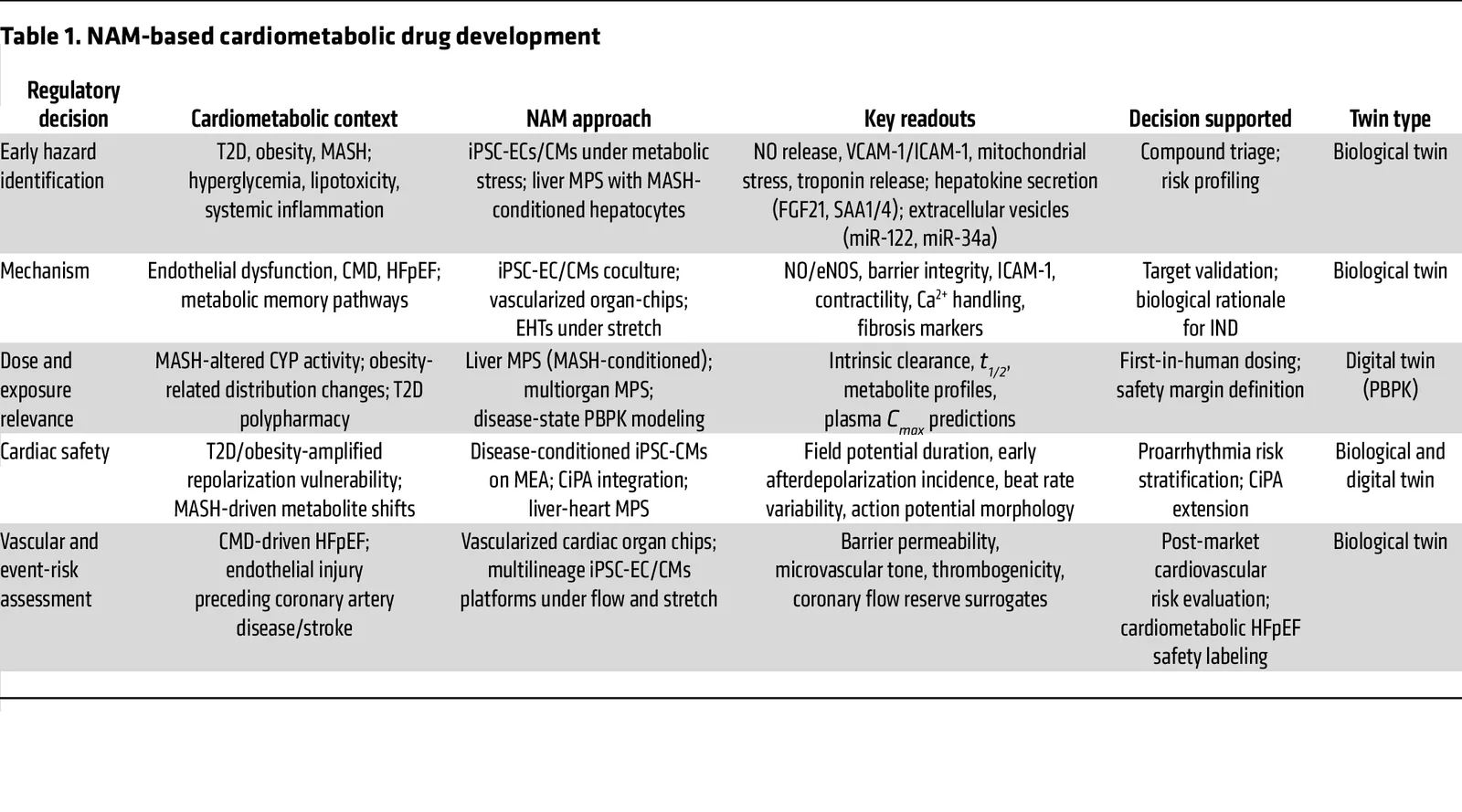
NAM-based cardiometabolic drug development

**Table 2:**
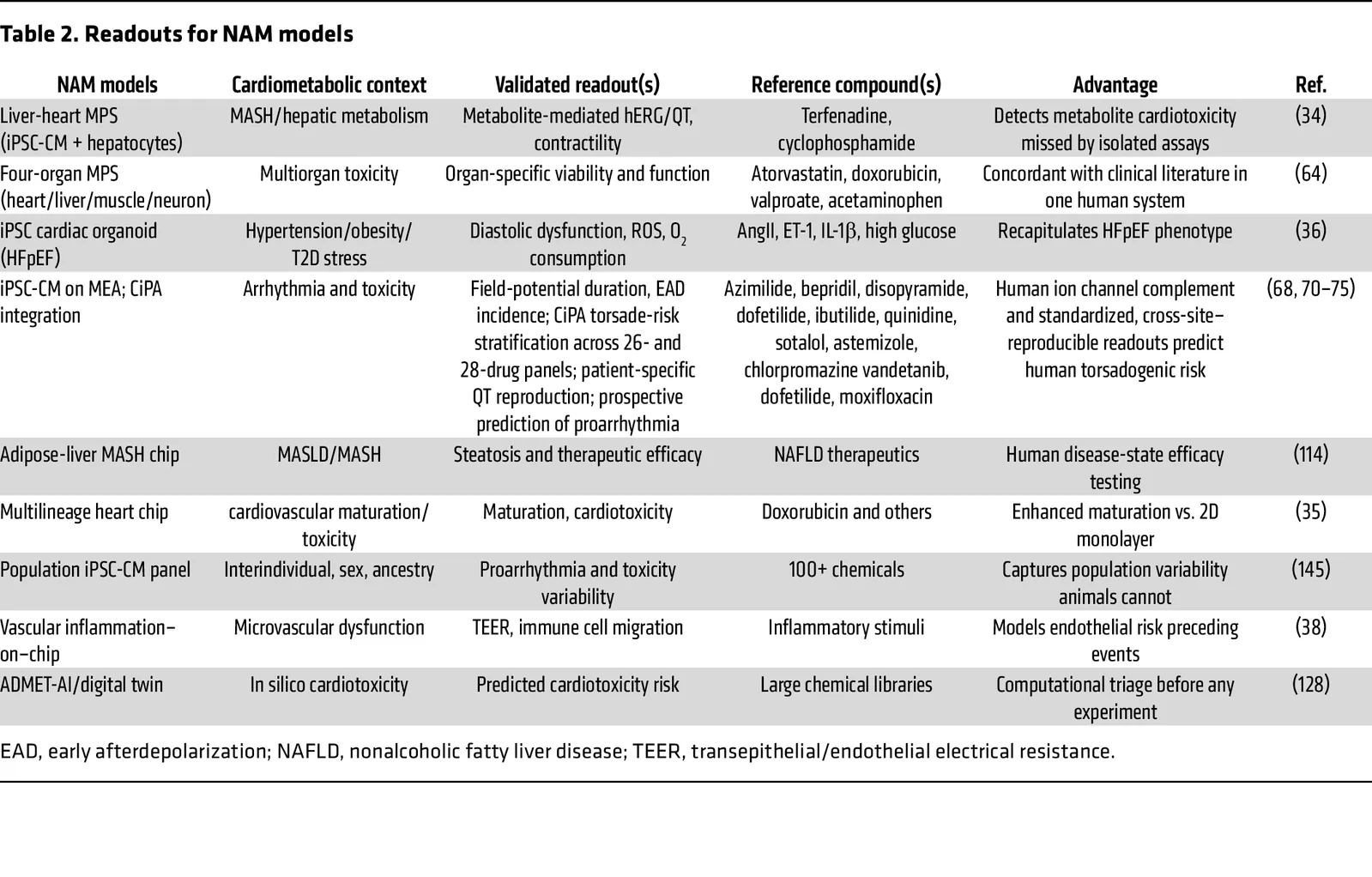
Readouts for NAM models

## Version 1 10/01/2026

Electronic publication
